# From Supernatural to Ornamental: Black Elder (*Sambucus nigra* L., Family Adoxaceae) in Sweden

**DOI:** 10.3390/plants13213068

**Published:** 2024-10-31

**Authors:** Ingvar Svanberg, Erik de Vahl, Navarana Ingvarsdóttir Olsen, Sabira Ståhlberg

**Affiliations:** 1 Swedish Institute for Russian and Eurasian Studies, Uppsala University, SE-75120 Uppsala, Sweden; luma.uppsala@gmail.com (N.I.O.); sabirien@pm.me (S.S.); 2 Department of Landscape Architecture, Planning and Management, Swedish Agricultural University, POM, SE-23422 Lomma, Sweden; erik.de.vahl@slu.se

**Keywords:** diachronic research, flower cordial, food plant, human–plant relations, urban ethnobiology, utility plants

## Abstract

Black elder, *Sambucus nigra*, is a non-native but now partly naturalized shrub in Sweden; it has been cultivated here at least since the Middle Ages. Previously, this plant was associated with a supernatural being to whom sacrifices were made, and its fruits were used in folk medicine and wood for fuel and crafts. Traditional economic uses vanished with industrialization and urbanization and black elder was mostly planted as an ornamental shrub in urban parks. At the end of the 1970s, however, it made a sudden comeback: city dwellers started to gather flowers to make a refreshing non-alcoholic cordial. This diachronic study of *Sambucus nigra* spanning over a millennium reflects various attitudes and uses within the context of a changing human society. In addition to the simple but popular cordial, side dishes and desserts made of its fragrant flowers are becoming increasingly popular in modern Swedish cuisine. Globally it has also been (re)discovered and the utilization of this plant is growing: its flowers are used to add flavor to soft drinks, salads, desserts and various dishes; berries are used for medicine and in cooking, especially with meats, and its future uses seem to be limited only by human imagination.

## 1. Introduction

### 1.1. Background

**Figure 1:**
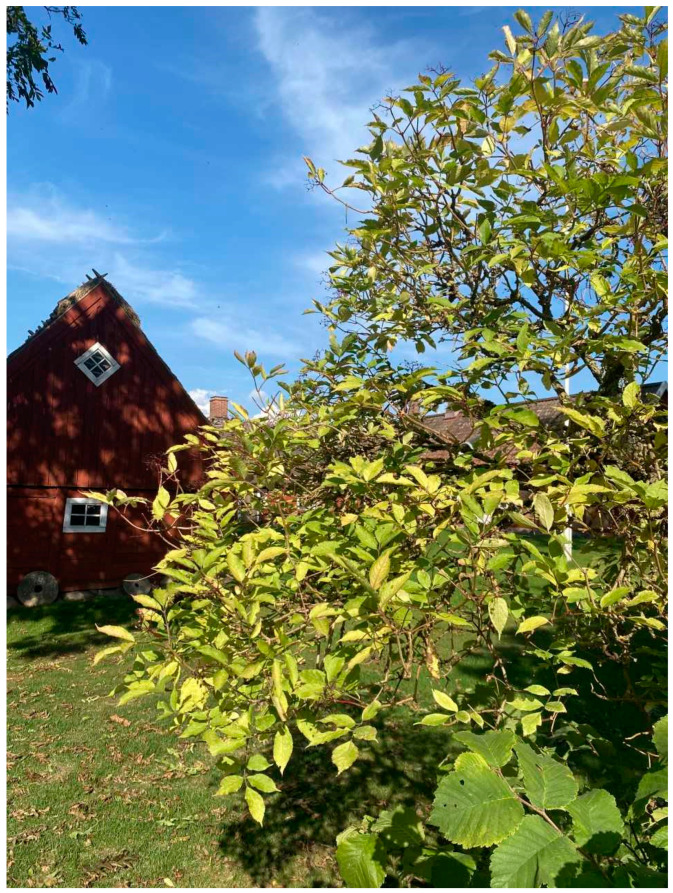
Shrub of black elder, *Sambucus nigra* L. in Skåne (Photo by Erik de Vahl, September 2024).

**Figure 2:**
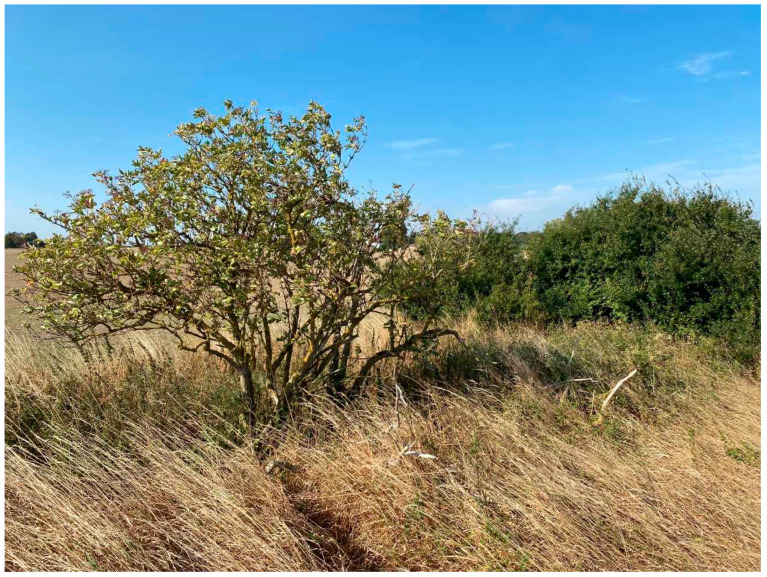
Naturalized black elder on Söderslätt, Skåne (Photo by Erik de Vahl, 2024).

Trees and shrubs are important elements in contemporary cityscapes: they contribute to biodiversity, provide multiple ecosystem services and influence the well-being of human and animal inhabitants [1]. Human–plant relationships naturally change over time and the processes can be observed through diachronic studies of specific taxa. Today, a multitude of trees and shrubs in urban parks throughout the world are common but non-native plants. This is also the case of a common shrub in Sweden, European black elder, *Sambucus nigra* L. Black elder was probably cultivated in Denmark already during Viking times (ca. 800–1050 CE) and from medieval times in southernmost Sweden, a part of Denmark until the seventeenth century [2] (Figure 1). Black elder has since become naturalized further north in Sweden, Norway and Finland, and it still maintains close links with human settlements and cultural landscapes (Figure 2).

This shrub became important for peasants in pre-industrial Sweden: in the south, it was associated with a supernatural being to whom sacrifices must be made. The fruits were used in folk medicine and its wood for handicrafts. Traditional economic uses disappeared during industrialization and urbanization at the end of the nineteenth century, and the function of black elder was reduced to an ornamental shrub in old gardens and urban parks. In the late 1970s, city dwellers suddenly began to collect its flowers in parks to make a refreshing cordial. Cordial (Swedish *saft*), as it is understood in Sweden, is a sweet fruit or flower-flavored drink or a drink made from fruits, berries or flowers and sugar. ‘Cordial’ in English usage could also mean an alcoholic drink, but the synonyms for ‘non-alcoholic cordial’, such as juice, squash, syrup, extract, nectar, etc., do not reflect the character of the Swedish elderflower drink. Therefore, we will use the term ‘cordial’ throughout this article, in the meaning of a non-alcoholic, refreshing fruit-, flower- or plant-based drink.

The new wave of popularity of black elder was connected with a growing interest in natural foods and home preparation of foods and drinks from wild plants, fueled by mass media and publications of fashionable recipes and cookbooks focusing on gathering food from nature. Parallel with black elder, other flower taxa also became popular for making lemonade, cordials and other products and dishes. Despite increasing competition from commercial soft drinks and foods with elderflower, including desserts and beverages not only produced by Swedish companies but also by global multinational giants, still fifty years later it is common to make elderflower cordial at home in Sweden, and the flowers are also used for cooking. This article discusses the cultural history of *S. nigra* in Sweden, its use as a cultivated plant in urban environments, urban harvesting and current and possible future uses of the flowers [3].

### 1.2. Biology

**Figure 3:**
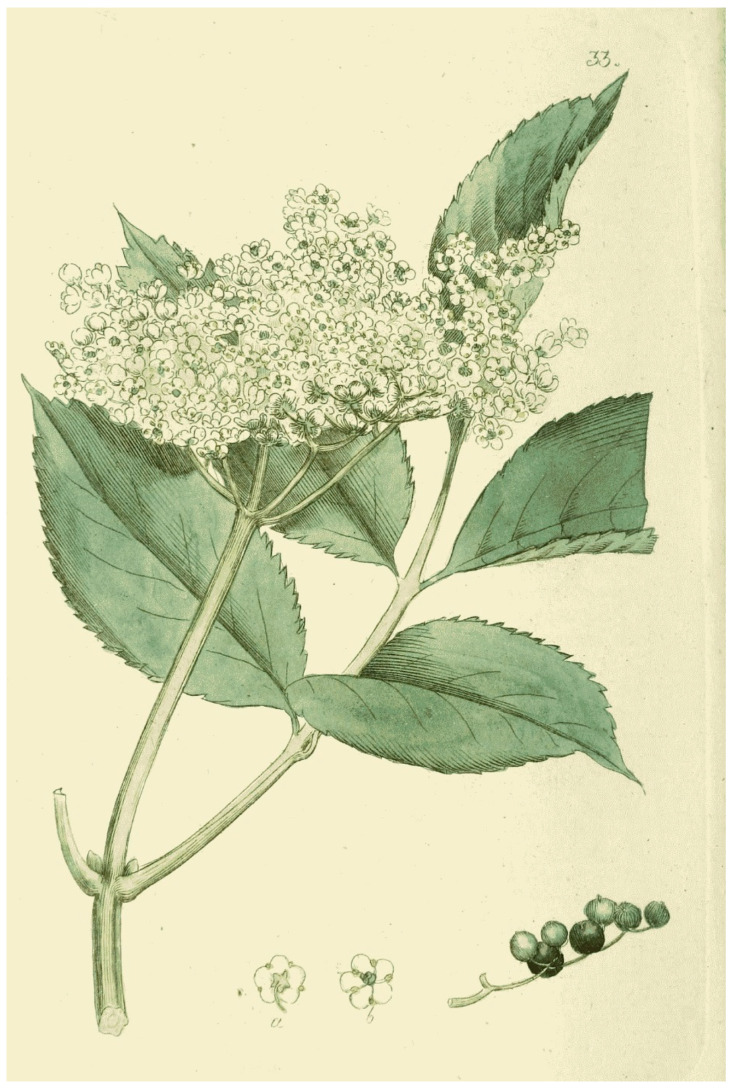
Flowerhead of *Sambucus nigra* L. (J. W. Palmstruch, *Svensk botanik* 1, 1800).

*Sambucus nigra* L. is a deciduous, large shrub, more rarely a small tree reaching up to 4–5 m; in Sweden it remains mostly a shrub due to the colder climate conditions. Straight, vigorous upright shoots grow from the base; branches and twigs have a pure white porous pith. It generally blossoms from May to July in Sweden, although variations occur depending on latitude and climate zone; the country extends for 1572 km from north to south. Black elder carries white flowers and bright yellow anthers set in broad, flat inflorescence; their scent is sweet and spicy. Elderberries are red at first but turn black and shiny when ripe, commonly in late September [3] (Figure 3).

According to the Swedish Poison Control Center, the flowers and ripe black berries are safe to eat. Ingestion of large quantities of berries, especially unripe ones, can however cause stomach cramps, nausea, vomiting and diarrhea [4], as the berries are mildly poisonous in their raw state, yet they are edible after cooking. Unripe elderberries and other parts of the plant contain essential oils and toxins such as saponins, and the leaves, bark, seeds and other parts of the plant contain considerable quantities of sambunigrin and other cyanogenic glycosides [5].

**Figure 4:**
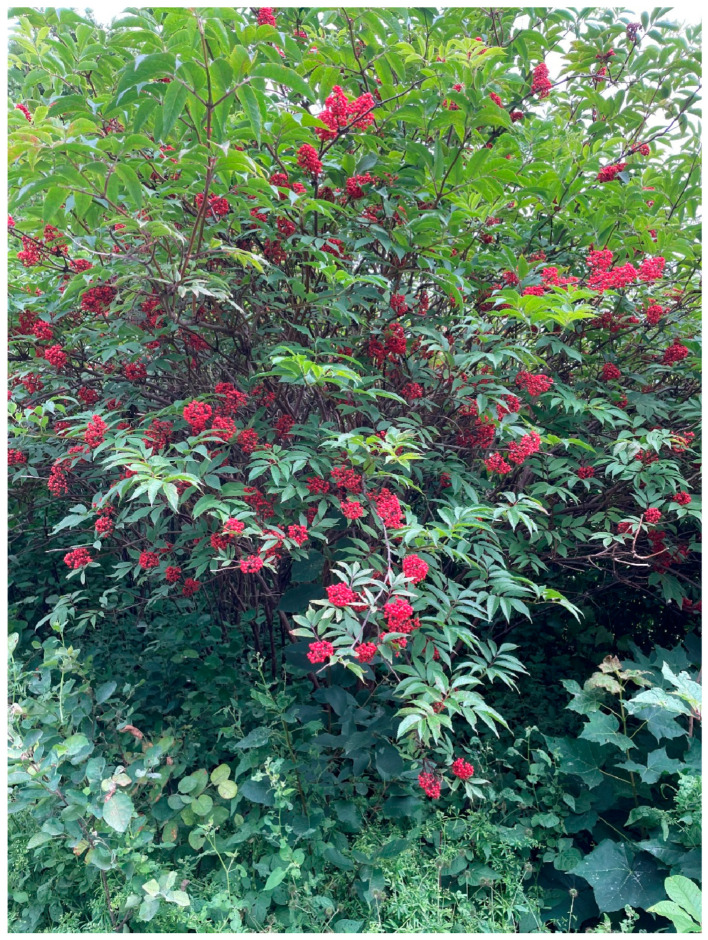
Red elderberry, *Sambucus racemosa* L., in Kapellgärdesparken, Uppsala, Sweden. This species was introduced to Sweden in the eighteenth century as an ornamental shrub in gardens and parks. Birds consume the fruits. The stem, root and foliage are considered to be poisonous and the fruits to be toxic to humans. Since red elderberry flowers bloom early, already in May, no one accidentally picks the flower heads, although newspapers warn readers every year (Photo by Ingvar Svanberg, 6 July 2024).

Black elder could be confused with red elderberry, *Sambucus racemosa* L. and danewort, *S. ebulus* L., both toxic. Red elderberry is also a shrub but has yellowish-white flowers in rounded bunches and red fruits; the pith of the branches is brown and it blooms earlier than black elder (Figure 4). Danewort is a large herb, around a meter high, with inflorescence and fruits like those of black elderberry but with red anther.

### 1.3. Distribution and Ecology

**Figure 5:**
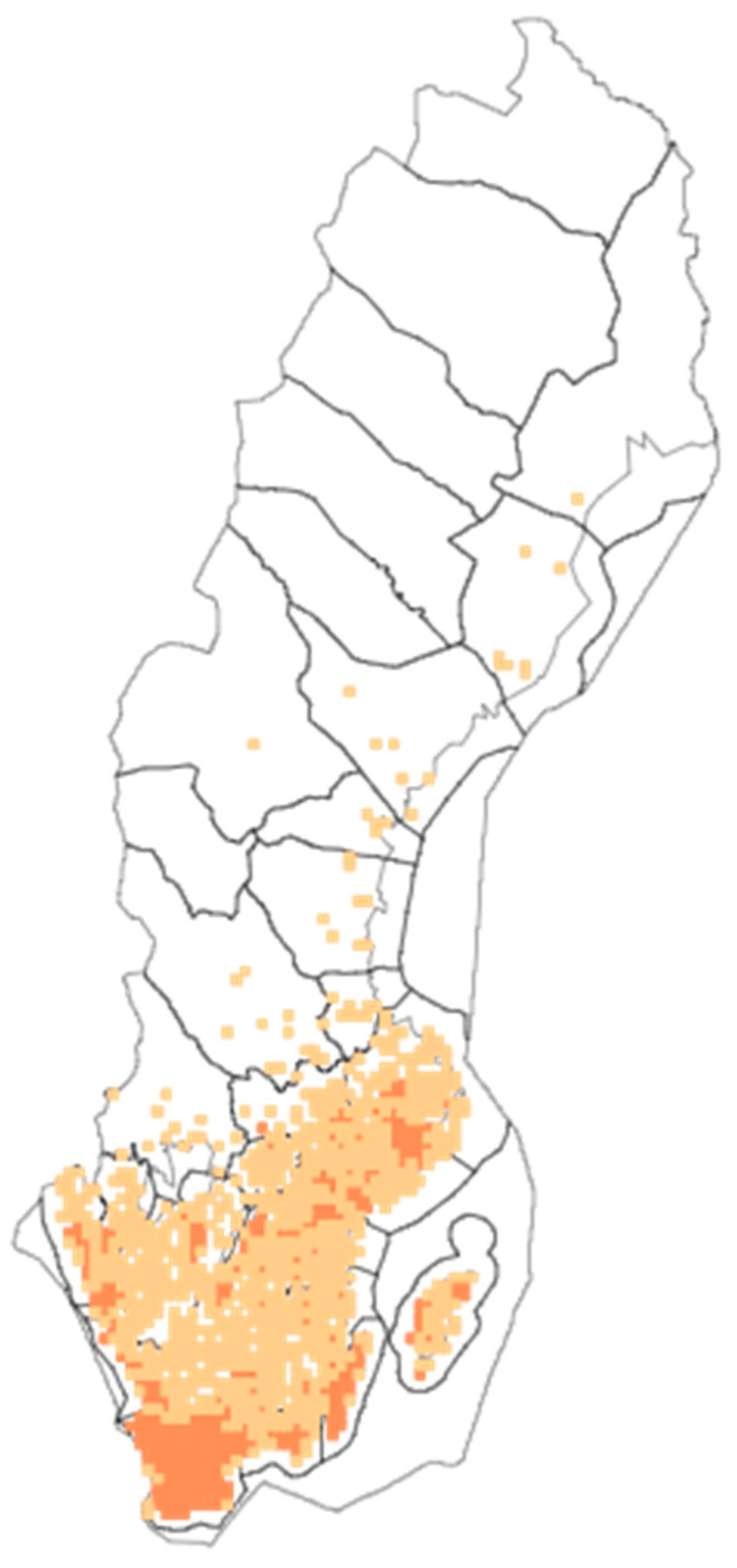
Distribution of *Sambucus nigra* L. in Sweden. Light squares < 10 observations, dark squares 10–500 observations (SLU Swedish Species Information Center).

Black elder is native to most of continental Europe but was introduced for cultivation to the Scandinavian Peninsula (Sweden, Norway) and Finland [3], where it has become naturalized: in Sweden, black elder can typically be found in woodlands in the southwestern part of the country and as a relic of earlier cultivation up to central Sweden and southern Norrland in the north [6,7] (Figure 5). Its presence in the wilderness often indicates a previous location of a garden or crofting sites. Black elder is planted in parks and around homes, in Sweden at least up to the province of Ångermanland, in Norway also north of the Arctic Circle. Birds spread elderberries effectively and, especially in southern Sweden, many occurrences are supposed to be from avian distribution. Black elder is commonly known as *fläder* in Swedish but in the south it is traditionally called *hylle* (cf. Danish *hyld*) [7].

### 1.4. Research Aim

The aim of this study is to discuss the history of the relationship between humans and black elder, urban harvesting of elderflowers and their use in the production of cordial and cooking. The role of black elder in Swedish folk botany is examined from a diachronic point of view. We also describe contemporary gathering activities and the production of cordial as a case study. The versatile use of elderflowers and possible future utilization are also discussed.

## 2. Results

### 2.1. Historical Context

There are few archaeobotanical data on black elder available from the Scandinavian peninsula; in Denmark, where it probably is native like in other parts of continental Europe, the earliest records of seeds date to the third and fourth centuries. The findings become more frequent during the Viking and Medieval periods [8]. There are no archaeobotanical findings from further north before the seventeenth century. In Skåne province in southernmost Sweden, black elder seeds from gardens have been discovered in the medieval city of Lund [9]. A find from an Iron Age settlement in the province is more uncertain. Skåne was part of Denmark from at least the ninth century until 1658.

In 1533, Canon (a kind of clergyman) Christian Petersen in Lund, then part of Denmark, mentioned remedies made of black elder, and Henrick Smid, a practitioner of medicine in Malmö, described them in 1546. Further north in Sweden, black elder is mentioned as medicine in a manuscript from the fifteenth century, and by Benedictus Olavi in 1578. In Finland, the easternmost province of Sweden until the nineteenth century, Mikael Agricola mentioned black elder in his prayer book from 1544 [10,11]. These written sources contain the earliest information about elder use, but the knowledge is probably copied from German or other continental sources; especially after Reformation, many Nordic students went to study at German universities.

### 2.2. Cultivation History

According to Carl Linnaeus, writing in the eighteenth century, black elder could be found in gardens from southern Sweden all the way up to the province of Gästrikland. He assumed that it was first introduced together with Roman Catholicism around the year 1000, although there are no indications that this was the case [12]. Archaeobotanical findings and pollen analyses show that it was certainly cultivated near settlements during the Middle Ages in Skåne, yet possibly introduced earlier [13]. After Skåne became a Swedish province in the mid-seventeenth century, black elder was probably planted on a larger scale throughout Sweden (including Finland) and neighboring areas such as Estonia and Norway.

Black elder bushes served mainly as a medicinal plant for peasants in pre-industrial Sweden and were therefore grown in villages. Johan Fischerström reported from Mälardalen in the eighteenth century that it grew “around villages and farms, always in such quantities that one could make mash from the berries at least for domestic use” [14]. Linnaeus observed in 1741 on Öland Island in the Baltic Sea that black elder was found in most villages [15]. A few years later, he noticed in Skåne that black elder bushes grew very abundantly in villages [16]. Linnaeus obviously did not like the shrub: its scent and taste were abhorrent and these signaled to him that it was poisonous. He believed that the flowers and berries killed chickens; his peacocks had been killed by the toxic berries [17]. Botanist Anders Johan Retzius noted that most elderberry bushes, except in southern Sweden, died during the harsh winter of 1740; many elderberry bushes also froze in southern Skåne, but the species quickly recovered through seed dispersal in bird droppings [18]. 

Black elder cultivation by peasants, crofters and vicars was small-scale (single bushes) and it was carried out close to the houses. The bush was grown chiefly because the berries were used for medicinal purposes, according to accounts from various parts of Sweden [19,20]. A record from Västmanland tells that black elder used to be found at almost every farm [21]. Yet, no old cultivars could be found during the call made by POM in 2010, probably because the traditional cultivation had already fallen into oblivion. The specific call for local cultivars selected for cultivation probably also excluded informants with knowledge of extensively cultivated and harvested elder.

In the first half of the twentieth century, black elder bushes were rarely planted and they appear to have indeed been forgotten. Instead, they were left to stand as cultural relics in old gardens and parks, or naturalized [22]. In the 1970s, however, black elder became common again, this time as a decorative park shrub, not least because it flowers profusely during the summer months when many other shrubs and trees already start to set fruit. Nowadays, black elder is both a popular park shrub and garden plant, admired, enjoyed and appreciated by most inhabitants in Sweden both in urban and rural environments.

### 2.3. Home of a Supernatural Being and Other Folklore

In many areas in Sweden, it was believed that the elder bush was inhabited by a mythical female being or protective spirit called *hyllefrun* ‘the elderberry lady’. She was a kind of *genius loci*, worshiped by the peasantry and sacrificed to; she had to be treated very well and provided with gifts. It was customary to make offerings to her on special occasions and feasts like Christmas. The elderberry lady was helpful but if one insulted her, she took her revenge with a red rash known as *hylleskåll* ‘elder scald’ appearing on the arms, hands and face; perhaps a kind of allergic reaction to the toxins in the bush. Retzius observed peasants with rashes all over their bodies several times in Skåne; they had slept under elder bushes [18]. Ethnologist Eva Wigström quotes a female informant in Skanörs Ljung: “If you have ever offended an elderberry lady and got an elder scald, or if a child has received an elder scald through the fault of its mother, then you should boil elder bark in milk, wash the wound with the liquid and then beat out the milk at the root of any scald”. In Kivik, one could suffer from elder plague “if one urinated or sat on an elder bush […]. It was then blown through elder pipes, nine in a row”. Wanting to pick the flowers for making tea, one had to say “*Hyllemor* [‘Elder mother’], *hyllemor*, may I take some of your flowers, and I will give you just as good flowers again”; only after that, one should start picking the flowers [23]. The black elder also had some negative connotations among the peasantry. A local historian in Skåne in the 1880s recalled that the old men refused to help him remove a self-seeded elderberry growing at the foundation of his house [24].

Elder twigs could be used for magical purposes since they were credited with apotropaic properties. Young women in Småland could thus protect themselves from violent husbands [24]. On Gotland Island in the Baltic Sea, black elder was a calendar sign. Ethnologist Nils Lithberg observed that when the black elder bloomed, local people thought that the fish around the island was at its best: this was the case from the end of July until August and the actual time for drift fishing [25]. 

### 2.4. Medicinal Plant

**Figure 6:**
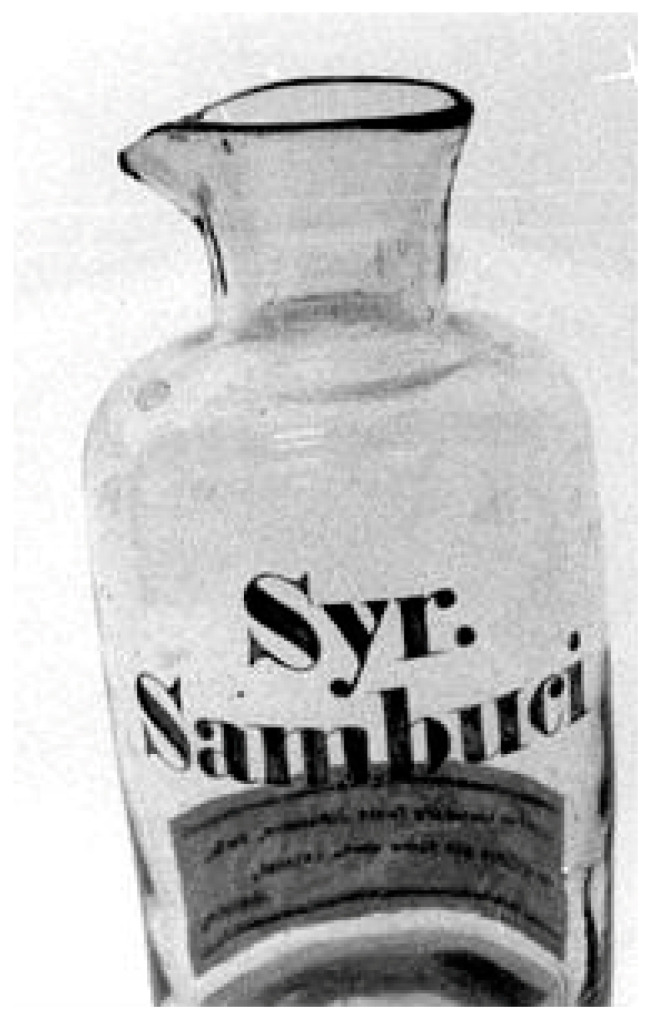
Bottle from Pharmacy “Lejonet” in Jönköping, Sweden. It contained Sambuci Syrup, made of sugar and elderberry, prescribed as a sweat expectorant (Courtesy Örebro Läns Museum, Sweden. CC BY-NC 4.0).

In continental Europe, black elder is a well-known medicinal plant, for centuries used to cure various illnesses, possibly due to its former folk religious status as a tree with special powers [3,26]. Elderberry remedies were available in pharmacies in Sweden as well and apparently highly popular: in the eighteenth century, Linnaeus and other natural scientists believed that the domestic consumption of elderberries could be met by cultivation in southernmost Sweden instead of through (the more expensive) imports. Black elder is already mentioned as medicine in a Swedish-language late medieval medical book, and it was included in the *Pharmacopoea suecica* ‘Swedish pharmacopoeia’ from 1775 until 1908. The drugs were imported by Swedish pharmacies from Germany and included *Sambucus cortex* (bark)*, Sambucus folia* (leaves)*, Sambucus flores* (flowers)*, Sambucus baccae* (berries), *Sambucus semina* (seeds) and *Sambucus syrupus* (syrup). Linnaeus noted that *Sambucus* was used externally as a repellent and also as panacea and sweat inducer. The bark has purgative properties [17] (Figure 6). In Dalsland and Värmland, an ointment for burns was prepared from the fresh middle bark of black elder and unsalted butter [27].

Today, the flowers are used mainly to make tea, desserts and cordial, but earlier, they could be used as warm wraps against colds: “Elderflowers are boiled, wrapped while they are hot in curtain fabric and placed around the neck”, stated August Bondeson [28]. The flower heads were traditionally used as a herbal tea, known as *fläderte* or *hyldete* ‘elder tea’. It was recommended as reducing fever already in the eighteenth century and still used for various ailments in homes in the early twentieth century [17,29]. Elder syrup, mashed elderberry and other products were also used in medicine until a century ago [30]. Mashed elderberry is mildly laxative and elderflower tea was the only remedy for colds in many households [31]. In the early twentieth century, elderflower tea became something of a fashionable drink and it is today widely available in health food stores in Sweden and many other countries.

### 2.5. Handicraft and Toys

The hard and yellow wood of black elder was very much sought after for handicraft and making all kinds of objects [17]; among other things, it is reported to have been used for smoke pipe stems. Retzius lists a number of tools made from elderberry wood, including weaving spoons, shoe-maker’s pegs and pulleys [18]. 

**Figure 7:**
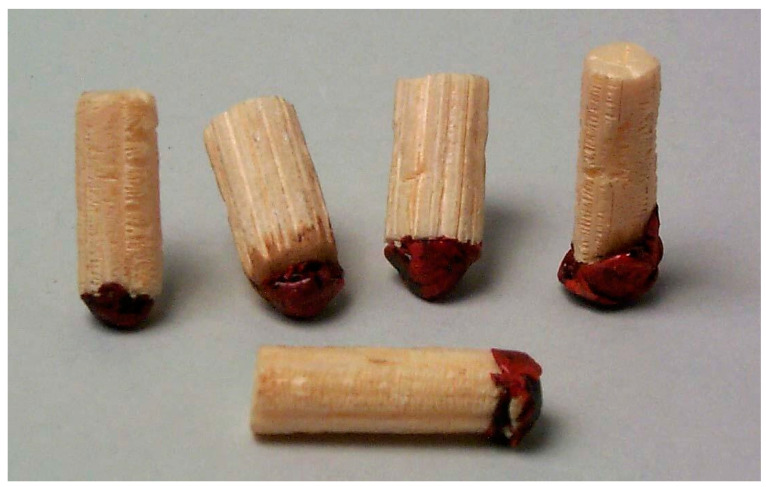
Toys called *flädergubbar* ‘old men’, made of the pith of elder, shot shells and red lacquer. By the weight of the shot shells, the old men always stand on their “heads” (Courtesy Gotlands Museum, Visby Sweden. CC BY 4.0).

A toy figure was made from elderberry pith, *flädergubbe* ‘elder oldie/old man’ or *trollgubbe* ‘troll oldie’, attached to a lead hemisphere. Because of the heavy lead, no matter how the ‘old man’ is placed, it always rises up, and after many bends and tilts, finally stops in an upright position (Figure 7). A blowpipe, known as an *fläderbössa* ‘elder gun’, has a wooden tube from which the pith has been removed. This toy is mentioned in sources already in 1749 and was known in the provinces of Skåne, Småland, Östergötland and Västergötland [32].

### 2.6. Miscellaneous Uses

Ripe elderberries were used for dyeing linen fabric to a dark brown color. The wood would be used as fuel, and leaves and branches could be hung up as repellents to drive away vermin from dovecotes and household utensils [33].

### 2.7. Ornamental Shrub

**Figure 8:**
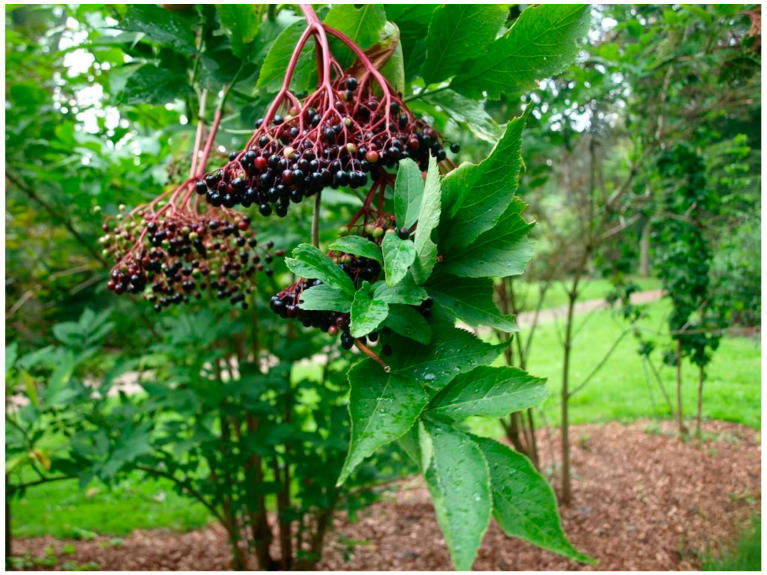
Black elderberry as an ornamental shrub in Alnarp Park, Skåne (Photo by Erik de Vahl, September 2024).

Black elder is today highly valued as a park plant because of the fullness and deep greenness of its foliage and the splendor and fragrance it develops during the flowering period (Figure 8). It is probably only surpassed by lilacs in popularity in Swedish parks. Some decorative cultivars are available in Swedish nurseries, including ‘Aurea’, ‘Black Lace’, ‘Bålsta E.’ and ‘Madonna’. Swedish breeding targets have not focused on the quality of the fruit (berry) but at finding a hardy provenance for seed propagation. 

Fruit trees, berry bushes and plants with edible parts make urban green spaces attractive to city inhabitants and visitors: ecological, social and economic values and significances are nowadays important factors in park planning. Growing these types of plants contribute to biodiversity, well-being and a healthier life, and induce more interest in and knowledge about plants and trees and how food is produced and grown. In the 1970s, some cities in Sweden introduced fruit trees, berry bushes and other plants with edible parts in parks. First, it became a fashion and then common to harvest fruits, among them elderflowers, and also of others such as blackthorn, *Prunus spinosa* L., which is prepared into a liquor. Harvesting occurred not only at the margins of the cities but also in the central parks of, among others, the central Swedish town of Uppsala in the 1970s and 1980s. Apples, plums and pears are still freely available in parks, although very few people harvest the fruits; a contributing factor might be fears about health hazards from air pollution and especially lead emissions. Also, rowan berries, *Sorbus aucuparia* L., are mostly left to the birds, and so are rose hips (*Rosa* sp.) [34].

### 2.8. Innovative Food

**Figure 9:**
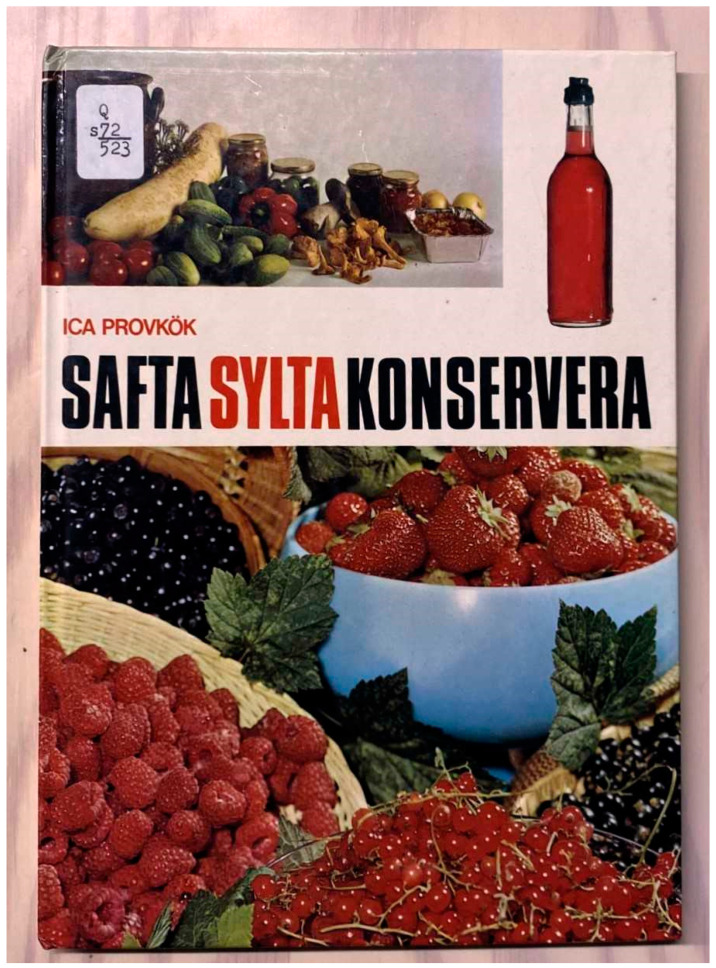
Britta Olsson’s book for ICA’s (supermarket chain in Sweden) test kitchen was published in 1972 and contained the first known published Swedish recipe of elderflower cordial. The same recipe with small variations conveyed through hundreds of newspaper and weekly magazine articles are still used by thousands of Swedes for home-made elderflower cordial. The book was also published in Danish in 1973 and has been republished several times in Sweden since the 1970s (Photo by Erik de Vahl).

**Figure 10:**
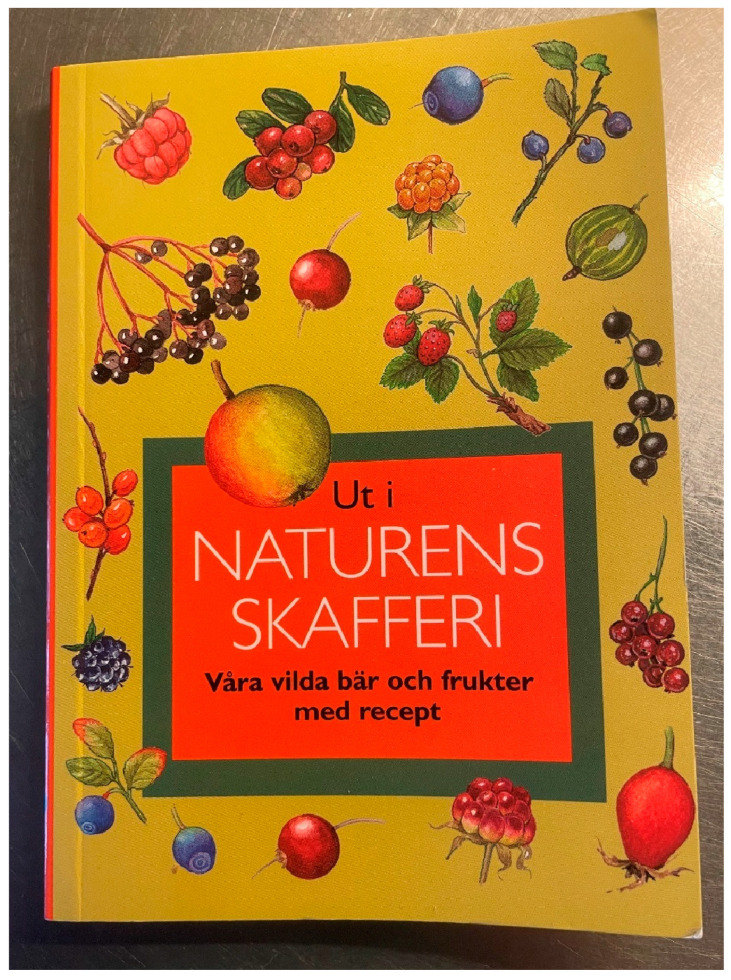
Booklet published by *Svenska Sockerbolaget* (Swedish Sugar Company, Malmö, Sweden) in 1998, with recipes made of fruits, berries and flowers. It was distributed free of charge in Swedish grocery stores. The booklet contains several recipes with elderflowers, elderberries and unripe elderberries (Photo by Ingvar Svanberg).

Elderflower tea was a common drink until the early twentieth century in Sweden, but it has today been replaced by elderflower cordial; in continental Europe, elderflower tea continues to be largely available. A book from 1969 by a well-known enthusiast about harvesting wild berries and fruits did not mention cordial made of black elder flowers [34]; the first recipe in modern times in Sweden dates back to the early 1970s. A recipe appeared in Britta Olsson’s famous food book in 1972 [35] and was reprinted in several newspapers the same year (Figure 9). This popular book had an immediate effect on elderflower usage: a review of extensive newspaper materials shows that basically the same recipe is reproduced in national newspapers every early summer since 1974. One example is *Göteborgs Tidning*, who wrote on 18 June 1976 that there are plenty of recipes for elderflower cordial. Since the 1980s, black elder recipes published in newspapers continue to develop and today extend far beyond the cordial. Elderflowers are included in recipes for pastries, salads, main courses, desserts, drinks, teas, sweets, ice-creams, pickles, etc. Both national and local newspapers feature a rich palette of elderflower cordial and other recipes every harvesting season (Figure 10).

#### 2.8.1. Elderflower Cordial

By far, the cordial surpasses the popularity of all other products made of black elder in Sweden. Black elder bushes are vastly available in city parks and little effort is required to gather the necessary number of flowers. The price of sugar is modest for an otherwise affluent population and the simple procedure of preparation contributes to the interest in elder cordial preparation among urban inhabitants. On the internet, thousands of gatherers share their experiences and information, tips and ideas about how to prepare cordial and how to use elder cordial for various dishes.

**Figure 11:**
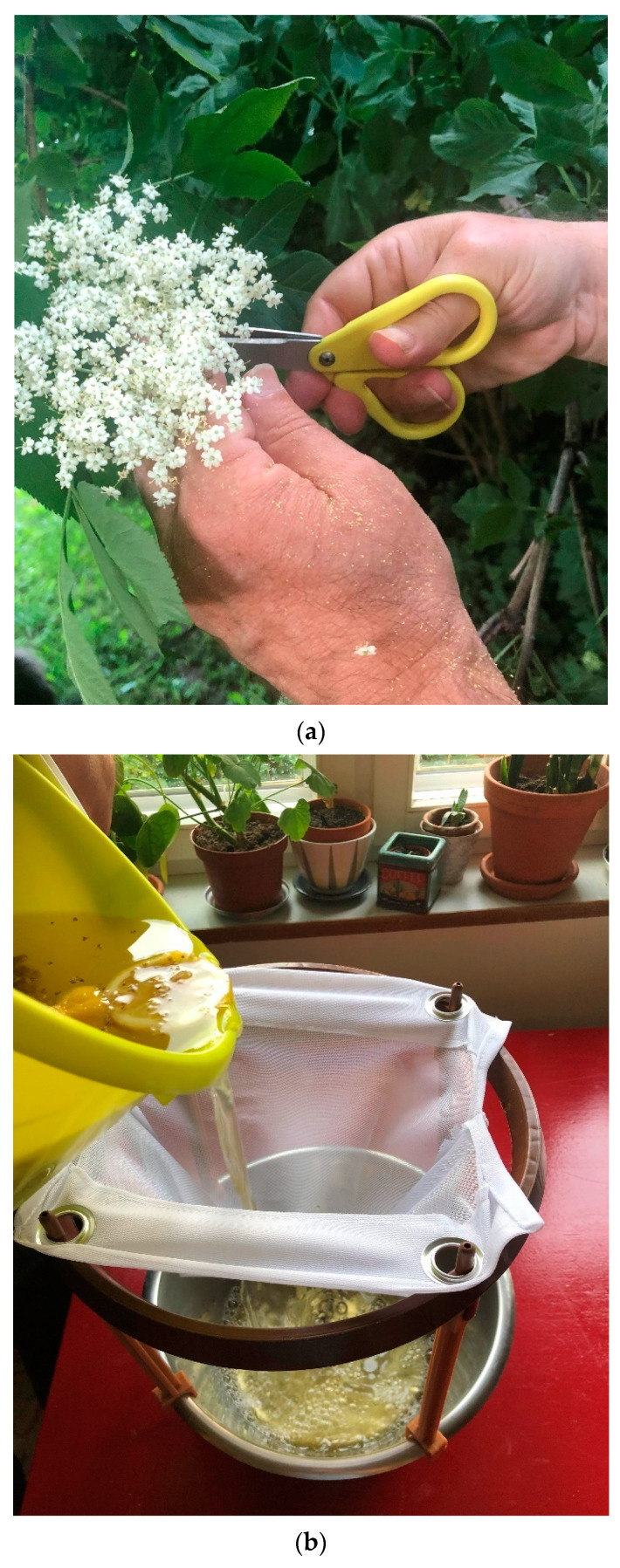

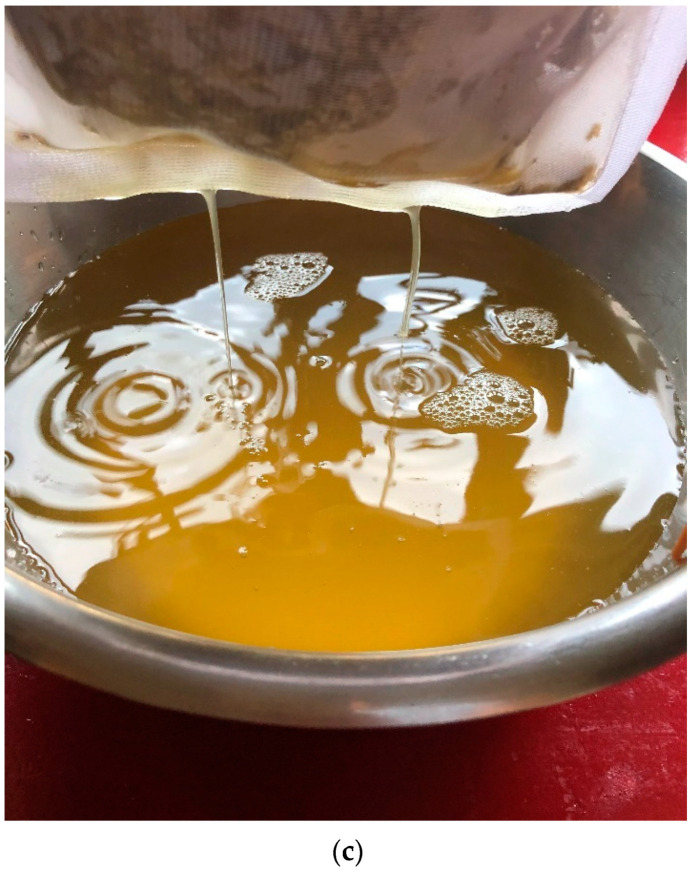
(**a**) Harvesting the flowers and making the cordial is a very simple procedure, neither time consuming nor costly. The flowers are easily harvested using scissors. After washing the flowers, hot water, sugar and lemons are added. The mixture is then left in the fridge for a few days. (Photo by Navarana Ingvarsdóttir Olsen, July 2020). (**b**) Pouring the elderflower mixture through a sieve is an important part of the making of cordial; the flowers and lemons are removed and the clear liquid can be bottled (Photo by Navarana Ingvarsdóttir Olsen, July 2020). (**c**) Squeezing the last drops from the sieve (Photo by Navarana Ingvarsdóttir Olsen, July 2020).

Making elderflower cordial is easy: 30–40 flowers are picked and elder aphids, *Aphis sambuci* Linnaeus, 1758, are washed off with water. The flowers are put in a bowl with 1.5 kg granulated sugar and 50 g citric acid. Three lemons are cut into thin slices and added; then 1.5 L of boiling water is poured onto the mixture, which is stirred until the sugar dissolves. After three days, the flowers are strained through a sieve and well-cleaned bottles are filled with the drink. If the bottles are plastic, they can be frozen (Figure 11a–c).

#### 2.8.2. Commercial Production

**Figure 12:**
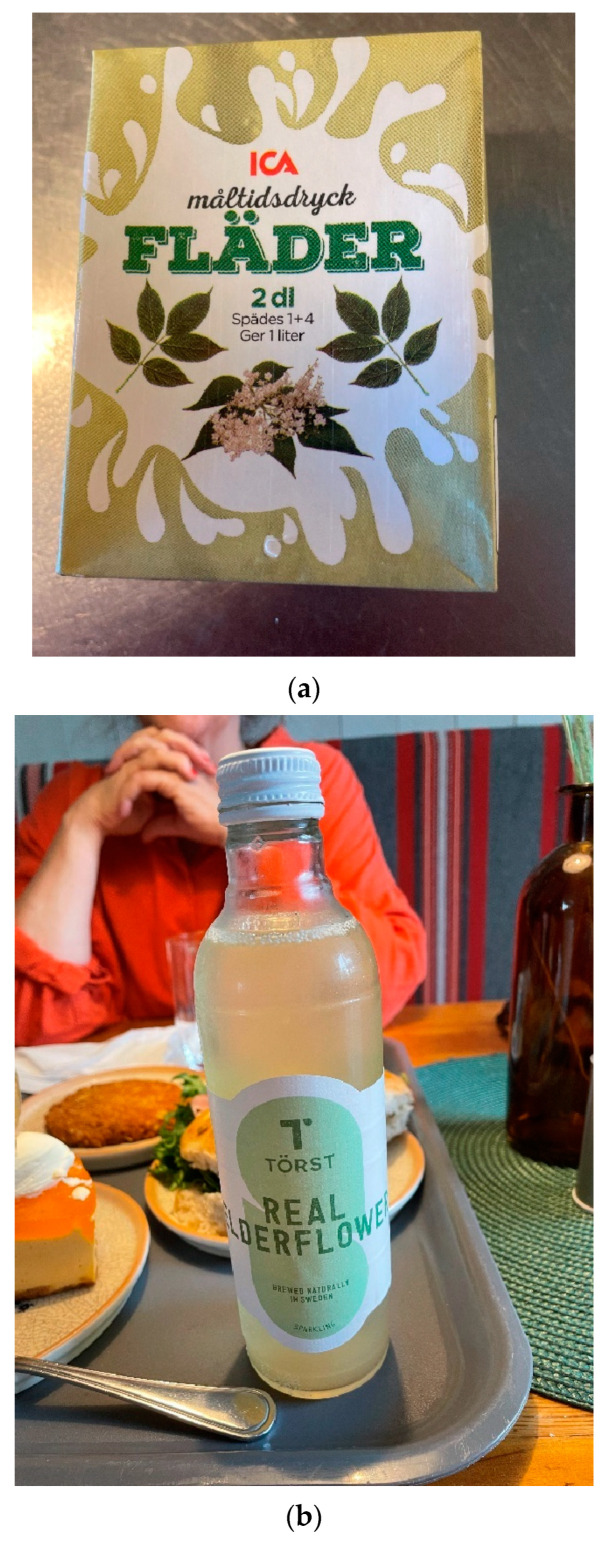

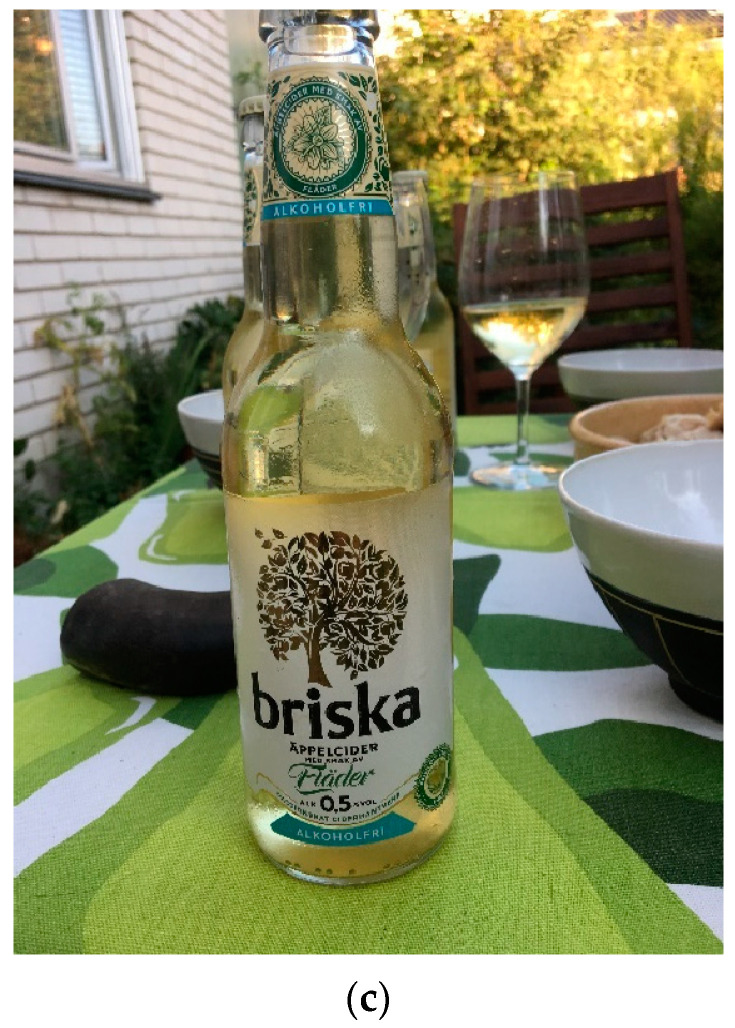
(**a**) Many commercial products made of or flavored with elderflower cordial are available in Swedish grocery stores. This drink is made of apple juice from concentrate, sugar and elderflower extract. The concentrate has to be diluted with water before drinking (Photo by Ingvar Svanberg, September 2024). (**b**) Törst ‘Thirst’, a refreshing non-alcoholic soft drink made of elderflower. (Photo by Ingvar Svanberg, September 2024). (**c**) Apple cider flavored with elderflower (Photo by Ingvar Svanberg, July 2021).

In continental Europe, demand for different elder products is far higher than in Sweden. There are commercial plantations of black elder for the production of elderberry juice. Elders are grown on a commercial scale in at least Austria, Denmark, Germany, Hungary, Italy, Poland, Ukraine and the United Kingdom. Elderflower wine is frequently mentioned in the English literature as a drink prepared by old ladies, and it is still popular in Britain [3,36]. The berries are used for coloring fruit juices and for making elderberry wine, juice and jam [37], and flowers are used for elderflower cordial, tea and wine [36]. In Sweden, only small-scale commercial cultivation occurs, for instance, *Flädergården* (yearly harvest 300 kg of flowers) in Vinslöv, Skåne, and *Lasätter fläder* (250 kg of flowers) near Nyköping in Södermanland. The government-owned spirits company Systembolaget produces a schnapps, *Halländsk fläder*, seasoned with elderflower cordial. Those who prefer not to prepare the non-alcoholic cordial or other products at home easily find elderflower cordial (mostly concentrate, to be diluted with water) in supermarkets; Swedes also receive cordial as gifts from friends or buy online from home producers. No adulterated black elder products have been reported in Sweden (Figure 12a–c).

### 2.9. Contemporary Multi-Purpose Use

The respondents to our questionnaire in 2019 reported that elderflowers were highly popular among harvesters of wild and park plants: the flowers were mainly used for non-alcoholic cordial, tinctures, schnapps, tea, ice-cream and wine; mixed with sparkling water, sparkling wine or hard liquors, elderflower cordial makes an excellent party drink.

Elderberry products are more popular than cordial among consumers on the European continent, but the making of elderflower cordial is far from unique to Sweden; it has also been reported at least from Denmark, the Faroe Islands, Finland, Germany, the United Kingdom, Spain and Italy. The preparation of cordial does not seem to be very old anywhere: it seems that it appeared in other countries around the same time as in Sweden. There are several causal links, including information and recipe sharing in newspapers over national, language and cultural borders, and the international popularization of TV programs on nature, food from nature and cooking shows intensifying and gaining large audiences from the 1970s onward. In the UK, making alcoholic cordial may be a much older tradition: home-made elderflower wine is mentioned as early as the mid-nineteenth century [38,39,40].

In Denmark, the cultural significance of elder has always been greater than in Sweden. Black elder was used for the same purposes as in Sweden, but it has a much wider use as food, for curing illnesses, handicrafts, toys, in folklore and in a number of linguistic expressions [10]. Contrastingly, in Norway, it has little significance [41]. In Finland, where black elder is cultivated in the southern part, preparation of cordial has recently become popular, especially among the Swedish-speaking minority, who closely follow media, events and fashion in Sweden. There are even small commercial companies in the Swedish-speaking Åland Islands and the town of Kerava/Kervo north of Helsinki who produce soft drinks and cordial [42].

The popularity of elderflower cordial has inspired experiments with other aromatic flowers and cordials in Sweden. This is a synergy effect of the acceptance of elderflower as food: the same recipe with sugar and lemon is used to make other flower cordials as well. In answers to our 2019 questionnaire, we received the following examples for making cordial: flowers from fireweed, *Chamaenerion angustifolium* (L.) Holub., meadowsweet, *Filipendula ulmaria* L., scented flowers of lilac, *Syringa vulgaris* L., and English dogwood, *Philadelphus coronarius* L. One informant in the northern province of Norrbotten even made cordial from the flowers of Arctic bramble, *Rubus arcticus* L. Cornflower, *Centaurea cyanus* (L.), is another flower that is utilized [43]. Many of these flower cordials can be bought at Medieval or Viking fairs, farmers’ markets, food festivals and similar historical and traditional theme events. Further suggestions for useful flowers, for example, bird cherry, *Prunus padus* L., can be found in great abundance on the internet, where many Swedes now search for recipes and inspiration. One cookbook suggests, among a multitude of species, to make cordial of the flowers of Turkish warty cabbage, *Bunias orientalis* L., an impressive but disliked weed in eastern Sweden [44]. Pastry chef and food author My Feldt recently published a book with the evocative title *Det växer saft och sylt överallt* ‘Cordial and jam are growing everywhere’, providing recipes for a great variety of flowers and berries [45].

In the agricultural, pre-industrial peasant society, wild plants did not play such an important role as is often imagined today. Wild plants were mostly used as emergency food, to supplement flour when crops failed or snacks for children; they were usually not part of the daily menu, until after sugar became cheap with the globalization of production, transportation and markets, for example, sour berries could be used more widely and became a regular part of the diet, often locally at first. Only in the urbanized and industrialized society of the twentieth century they became nationally known and consumed; today, many such wild products also find their way from marginal regions into international markets [43]. 

Making cordial from elder and other flowers is a step further and should be seen as products suitable for a post-industrial society, already distanced from nature and natural resources, but with a wish at least among a certain percentage of the urban population to return to their ”roots” or traditions of gathering and preparing food and drink at home. It is a case of urbanite nostalgia for the ”lost” rural life. For many, it is a hobby to gather and prepare home-made products, comparable with gardening, recreational fishing, hunting or keeping pets; collecting wild and park plants and fruits connects them with other foragers and creates social and informational exchange; internet forums function as their main meeting places.

### 2.10. Future Prospects

**Figure 13:**
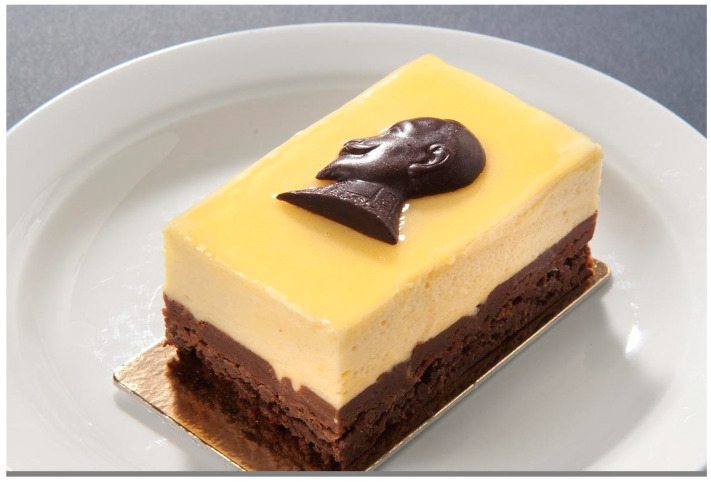
‘King Gustavus Adolphus pastry’, baked by a pastry shop in Stockholm for the national celebration on 6 November 2012, with cream of elderflower cordial and the king’s (ruled 1611–1632) portrait in chocolate (Photo by Peter Sergermark/The Nordic Museum CC BY-NC-ND).

Elderflower cordial and other elder products have great potential for development. Innovative chefs and confectioners are creating recipes for restaurants, cafes and patisseries; cooks are preparing newspaper and magazine articles and food recipes, as well as cooks on TV programs and video channels; and thousands of home cooks are exploring and creating new ways of using elder products. Elderflower cordial is already included in recipes for pastries, desserts, ice-cream, drinks, pickles, sweets, flavored mineral water, soft drinks, marmalade, quark, cheesecake, granita, tiramisu, parfait, punch, pancakes, pickled herring and many other foods and drinks (Figure 13). It is an excellent ingredient for a large number of dishes of various kinds and it seems that the only limit for its utilization is human imagination.

## 3. Materials and Methods

### Data Collection and Analysis

The relationship between humans and biota is complex; therefore, ethnobiological studies normally include a varied and broad set of methods for gathering data [46]. Methodologically, this study is in line with the source pluralism method of agrarian history and historical ethnobiology [47]. Our methodological approach is inspired by modern cultural history and rich empirical narratives; the analysis is qualitative. Sources of various character were used for data collection:

(1) Qualitative questionnaires for documentation of information about everyday life and plant use. Data gained from questionnaires sum up respondents’ experiences, beliefs and memories, and offer understandings which are of great value when gathering data, especially about present-day foraging activities. In 1956, the Dialect and Folklore Archive in Uppsala sent out a questionnaire called “Management and use of wild trees, shrubs and herbs in inland areas” [48]. Our study aims to shed light on contemporary foraging activities among urban dwellers in post-industrial Sweden; therefore, a new questionnaire with open-ended questions was designed and distributed mainly via social media platforms and online forums for foragers, such as *Vilda ätliga växter i Sverige* ’Wild edible plants in Sweden‘ (approximately 36,000 members, fall 2019), and also via the authors’ personal networks. Survey responses were collected during a limited time period between September and October 2019. A total of 81 answers were received and analyzed: the group of respondents consisted of 70 women, 7 men and 4 informants who did not classify their gender. A majority (*n* = 60) of the respondents lived in large cities in the southern part of Sweden. Most of the participants (*n* = 22) were born during the 1970s, 16 during the 1960s, 15 during the 1980s, 13 during the 1990s, 13 during the 1950s and 3 during the 1940s or earlier. A trend showing a growing interest in wild plants is visible. In contrast, when POM (The Program for Diversity of Cultivated Plants) at the Swedish University of Agricultural Sciences (based in Alnarp, Uppsala and Umeå) in 2010 called for information on local varieties or heirloom cultivars of *Sambucus nigra*, no answers came forth [49].

(2) For research on the process of acceptance and distribution of black elder cordial home production, we analyzed newspaper materials, including the rich corpus of digitalized newspapers found in the Swedish Royal Library database with almost 1600 newspaper titles from the seventeenth century until the present day [50]. Articles, reports and recipes on elderflower cordial are often published in the summer months. These reports are studied from a diachronic perspective in order to outline at what time in history the cordial and other uses of elderflower became accepted as food stuff in Sweden.

(3) Data from culinary books, scientific reports, ethnographic descriptions, botanical works and local historical studies. We searched online databases like PubMed and SCOPUS for comparative studies outside Scandinavia.

(4) Ethnographic methods by participant observation are also applied with elements of an autoethnographic approach, as all authors have collected elderflowers and prepared cordial, added flowers to dishes and consumed commercial black elder products for decades. Preparing and observing during preparation processes are essential when studying plant use, and for the understanding of urban dwellers’ interest in this and other park shrubs as food and drink [51].

## 4. Discussion

In contrast to continental Europe [38,39,52], harvesting elderberries to make juice has never become popular in Sweden [35,43,44]. Black elder was an important species in the agricultural landscape, propagated and dispersed by birds and extensively managed and used by humans. Peasants in pre-industrial Sweden kept single bushes and prepared mashed berries as a medical remedy, sacrificing and revering the bush as the home of a supernatural being. Home-made products such as tea made of the flowers were still used in homes at the beginning of the twentieth century, but they were soon replaced by imported and industrially produced medicines. The function of the elder bush was reduced to an ornamental park plant and in the post-war period, its popularity as a park shrub has grown [53]. Since the 1950s, recipes were published for making a kind of caper from its unripe berries but as these are somewhat toxic, these proposals never caught the public’s attention in Sweden. The product can still be found in local food shops and in recipes but it is fairly unknown [54,55].

The idea that elderflowers could be made into a non-alcoholic cordial caught on rapidly in the 1970s after the publication of a recipe and its distribution through media. The fact that elderberry bushes were already available in city parks, thus reachable for a majority urban population, contributed to the sudden fashion together with the little effort required to gather the flowers and the easy preparation process. Further, the price of sugar was low; also, an interest among urban dwellers toward ‘do-it-yourself’ food traditions and health issues was growing and has gained more speed ever since the 1980s. Cookbooks, TV shows and nature programs increasingly present edible food from nature and urban and rural environments, and today, many Swedes look for information on the internet, cookbooks and forums for modern urban foragers [56].

Home preparation of elder products had no economic significance for the informants in our 2019 survey, but the respondents enjoyed making home-made cordial and perceived it as a pleasant hobby: they felt good when able to produce something tasty and drinkable they could enjoy all year round. Swedish families often serve their cordial to guests, proud of their home-made products, and often bring a bottle as a gift when visiting. Many Swedes also associate the taste of elder cordial with summer and sunny memories.

This urban harvesting of elderflowers is an interesting example of a new relationship between humans and a shrub; it has developed only within a few decades. “It’s free and environmentally friendly”, explained one informant in our 2019 survey. “For me, it creates a sense of joy and independence”, emphasized another. “Free and locally produced”, said a third. For urban foragers, the ability to gather natural edibles is a free asset and city parks offer a kind of ecological compensation for people living far away from nature in a city environment. Harvesting edibles in the parks rather than in the forest increases plant accessibility for city dwellers. According to the Statistics Sweden (SCB), more than 88 per cent of the population in Sweden live in urban settlements in 2024 (https://www.scb.se/hitta-statistik/sverige-i-siffror/miljo/tatorter-i-sverige, accessed on 1 September 2022). Like many other foraging activities (mushroom hunting, gathering wild plants for food, etc.) in Sweden today, the harvesting of elderberry blossoms is the concern of the urban population, not a residual rural activity. The urban middle class especially shows a growing interest in gathering, home preparation of foods, visiting farmers’ markets and participating in food and historical festivals; therefore, urban harvesting in Sweden is increasingly encouraged by town and park planners.

City harvesting is an understudied topic, as are immigrant groups and foreign students, who collect wild and park plants in cities for consumption and medicinal purposes. Urban ethnobiology is a dynamic field of research, located at the intersection of what we usually call culture and nature [57,58]. It must necessarily involve new issues and interesting challenges for researchers from both theoretical and methodological perspectives. Among urban subcultural circles, there are groups who want to become more independent in their food production, moving away from commercial food production and processed foods, supermarkets and the risk of being dependent on a globalized market; health considerations are also important for many urban dwellers. For ethnobiologists, the field of urban studies offers exciting new (re)discoveries of old habits, emotional and sensual as well as multiple perceived and imagined relationships with plant materials, creative new opportunities and interpretations about the present time, traditions and history, and human relations with the surroundings, not only wilderness or rural but to a high degree—the urban.

## 5. Conclusions

This study shows that the relationship between humans and black elder, *Sambucus nigra* L., has been maintained since at least the Viking Age in southernmost Sweden and Denmark, and later in other parts of the Nordic countries, and how this relationship has fluctuated over a thousand years. Probably, the presumed medicinal properties of the elderberry led to its introduction to the Scandinavian peninsula. It was first connected with folklore and beliefs, medicine, creation of handicrafts, toys and other objects, and then planted for its ornamental qualities, until Swedish mass media distributed cordial recipes from the 1970s onward. Preparing elderflower cordial has since become a hugely popular activity practiced by both the old and young in the country. Today, restaurants, cafes, patisseries, cookbook and recipe writers and TV and video channel chefs are intensely searching for new edible plants. Elderflowers have been identified as a versatile ingredient and also home cooks experiment with adding the flowers to all kinds of dishes and drinks and publish their results on the internet. The use of elderflowers has already found many applications in everything from drinks to main courses, pastries and desserts, and new recipes are being published regularly online and in cookbooks. Despite commercial products such as soft drinks with elderflower available in shops and markets, home-made elderflower cordial holds its place in the hearts and fridges in Sweden.

## Data Availability

All data generated or analyzed during this study are available in this article.
